# Aquaporin 2 in Cerebral Edema: Potential Prognostic Marker in Craniocerebral Injuries

**DOI:** 10.3390/ijms25126617

**Published:** 2024-06-16

**Authors:** Wojciech Czyżewski, Jan Korulczyk, Michał Szymoniuk, Leon Sakwa, Jakub Litak, Dominik Ziemianek, Ewa Czyżewska, Marek Mazurek, Michał Kowalczyk, Grzegorz Turek, Adrian Pawłowski, Radosław Rola, Kamil Torres

**Affiliations:** 1 Department of Neurosurgery, Maria Sklodowska-Curie National Research Institute of Oncology, ul. W.K. 7 Roentgena 5, 02-781 Warsaw, Poland; 2 Department of Didactics and Medical Simulation, Medical University of Lublin, 20-954 Lublin, Poland; 3 Department of Plastic, Reconstructive Surgery with Microsurgery, Medical University of Lublin, 20-954 Lublin, Poland; jan.korulczyk@umlub.pl (J.K.); kamiltorres@wp.pl (K.T.); 4 Department of Neurosurgery and Pediatric Neurosurgery, Medical University of Lublin, 20-954 Lublin, Poland; michmatsz@gmail.com (M.S.); marekmazurek@hotmail.com (M.M.); rola.radoslaw@gmail.com (R.R.); 5 Faculty of Medical Sciences and Health Sciences, Kazimierz Pulaski University of Radom, 26-600 Radom, Poland; sakwus@gmail.com; 6 Department of Clinical Immunology, Medical University of Lublin, 20-954 Lublin, Poland; jakub.litak@gmail.com; 7 Department of Otolaryngology, Mazovian Specialist Hospital, 26-617 Radom, Poland; czyzewska.ewa@hotmail.com; 8 1st Department of Anesthesiology and Intensive Care, Medical University of Lublin, ul. Jaczewskiego 8, 20-954 Lublin, Poland; michalkowalczyk@vp.pl; 9 Department of Neurosurgery, Postgraduate Medical Centre, Brodnowski Masovian Hospital, 8 Kondratowicza Str., 03-242 Warsaw, Poland; turek.grz@gmail.com; 10 Department of Human, Clinical and Radiological Anatomy, Medical University of Lublin, 20-954 Lublin, Poland; avandare@gmail.com

**Keywords:** AQP, aquaporin, brain edema, AQP1, AQP2, AQP4, AQP9

## Abstract

Despite continuous medical advancements, traumatic brain injury (TBI) remains a leading cause of death and disability worldwide. Consequently, there is a pursuit for biomarkers that allow non-invasive monitoring of patients after cranial trauma, potentially improving clinical management and reducing complications and mortality. Aquaporins (AQPs), which are crucial for transmembrane water transport, may be significant in this context. This study included 48 patients, with 27 having acute (aSDH) and 21 having chronic subdural hematoma (cSDH). Blood plasma samples were collected from the participants at three intervals: the first sample before surgery, the second at 15 h, and the third at 30 h post-surgery. Plasma concentrations of AQP1, AQP2, AQP4, and AQP9 were determined using the sandwich ELISA technique. CT scans were performed on all patients pre- and post-surgery. Correlations between variables were examined using Spearman’s nonparametric rank correlation coefficient. A strong correlation was found between aquaporin 2 levels and the volume of chronic subdural hematoma and midline shift. However, no significant link was found between aquaporin levels (AQP1, AQP2, AQP4, and AQP9) before and after surgery for acute subdural hematoma, nor for AQP1, AQP4, and AQP9 after surgery for chronic subdural hematoma. In the chronic SDH group, AQP2 plasma concentration negatively correlated with the midline shift measured before surgery (Spearman’s ρ −0.54; *p* = 0.017) and positively with hematoma volume change between baseline and 30 h post-surgery (Spearman’s ρ 0.627; *p* = 0.007). No statistically significant correlation was found between aquaporin plasma levels and hematoma volume for AQP1, AQP2, AQP4, and AQP9 in patients with acute SDH. There is a correlation between chronic subdural hematoma volume, measured radiologically, and serum AQP2 concentration, highlighting aquaporins’ potential as clinical biomarkers.

## 1. Introduction

Traumatic brain injury (TBI) is a group of diseases considered to be a serious problem occurring globally in the medical sector, both in high- and low-developed countries [1], without unified protocols, guidelines, or best practices to guide practitioners in their daily practice. This has prompted ongoing efforts to enhance care through structured neurotrauma services and international collaboration aimed at improving outcomes across diverse healthcare settings [2]. The essence of TBI is tissue dysfunction induced by a cascade of mechanical stimuli, leading to disturbances in central nervous system (CNS) homeostasis [3,4,5]. The search for serum biomarkers has been initiated with the aim of significantly enhancing patient care and outcomes in traumatic brain injury cases [4]. These biomarkers would facilitate patient stratification, minimize unnecessary imaging, predict intracranial lesions, guide imaging decisions, enable early detection of axonal injury, determine return-to-play timelines, monitor treatment effectiveness, provide realistic prognosis information, assess outcomes through audits, and identify chronic neurodegenerative complications [6,7].

Despite ongoing efforts, no single biomarker has yet emerged to effectively support the diagnosis and monitoring of both acute and chronic subdural hematoma in particular, a common consequence of TBI [8]. In response to this gap, research has turned to investigating alternative biomarkers, including aquaporins (AQPs), which are a family of water channel proteins that play critical roles in maintaining water homeostasis in various tissues, including the brain.

Aquaporin-1 (AQP1) is expressed in the choroid plexus epithelium, where it contributes to the production and circulation of cerebrospinal fluid (CSF) and helps regulate intracranial pressure. Additionally, it is found in cortical neurons. It has recently been found that its expression is regulated by arginine vasopressin V1a receptors, which are implicated in the formation of post-trauma brain edema [9]. Aquaporin-2 (AQP2) is a water channel protein primarily found in the kidney, specifically in the cells lining the collecting ducts [10]. Its main function is to regulate water reabsorption, thereby controlling the concentration of urine [11]. Aquaporin-4 (AQP4) is predominantly found in astrocytes, particularly concentrated in their endfeet, facilitating bidirectional water movement in response to changes in osmotic conditions [12]. Aquaporin-9 (AQP9) was identified in tanycytes, astrocytes, dopaminergic neurons, and microglia, and it is hypothesized to be involved in neuroinflammation [13]. AQP1, AQP2, and AQP4 seem to play a role in the perception of pain [14,15].

Several studies have explored the role of AQPs in TBI, examining their expression patterns and correlations with radiological imaging findings. Specifically, AQP1, AQP4, and AQP9 have been implicated in the pathophysiological responses to brain injuries, serving as key modulators of water movement across cell membranes and contributing to the development of cerebral edema [16]. Nevertheless, no study has been found that directly correlates the expression of aquaporins, measured as the concentration of serum proteins, with radiological parameters assessed in patients with subdural hematomas (SDH). However, several studies have investigated AQP’s in other types of traumatic brain injury (TBI). 

Despite many observations made in the context of the role of aquaporins in TBI, the importance of aquaporin 2 remains incompletely understood [13,16,17]. At the outset of this study, there was no existing literature or research examining the expression levels of AQP2 in response to brain injury, in contrast to its counterparts, AQP1, AQP4, and AQP9. AQP2 is primarily known for its role in the renal handling of water, specifically in the regulation of water reabsorption in the collecting ducts of the kidney, which is markedly different from the brain-centric roles of AQP1, AQP4, and AQP9.

We hypothesized that the absence of evidence linking AQP2 expression changes to TBI underscores its potential utility as a control in experimental studies investigating the regulation and function of aquaporins in brain injury contexts.

The objective of the presented study was to investigate the potential of brain aquaporins (AQP1, AQP4, and AQP9) as biomarkers for traumatic brain injury (TBI) and to assess AQP2 as a control group due to its hypothesized stability in serum concentration in TBI patients. Additionally, we aimed to compare differences in serum concentrations of brain aquaporins at various time intervals with the presumed constant serum level of AQP2.

## 2. Results

### 2.1. Patient Characteristics

**Figure 1:**
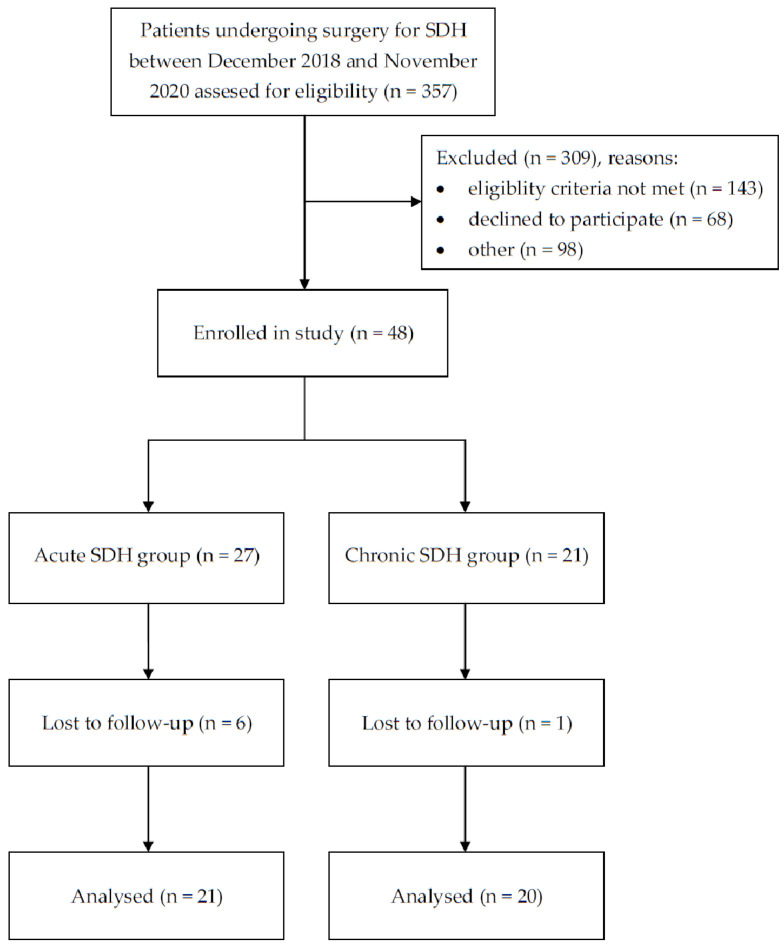
Patient flow diagram.

A total of 41 patients were included in the current study. Figure 1 shows a detailed participant flow diagram. A total of 21 of the study participants were diagnosed with acute SDH (aSDH) and 21 of them with chronic SDH (cSDH). The mean and median patients’ ages were 65.7 and 64 years old, respectively. The study included 31 male patients and 10 female patients. The median Glasgow Coma Scale (GCS) score reached 14 (IQR: 3). According to the neurological examination performed in each study participant preoperatively, 24 patients were presented with paresis, anisocoria was observed in 7 study participants, and speech impairment was present in 22. Based on preoperative CT scans, the median hematoma volume was 12 cc (IQR: 90), the median midline shift reached 8 mm (IQR: 8), and the median hematoma density amounted to 40 HU (IQR: 29). The median plasma aquaporin concentrations measured before surgery were 2.81 ng/mL (IQR: 0.79), 3.60 ng/mL (IQR: 1.38), 1.394 ng/mL (IQR: 0.56), and 2.47 ng/mL (IQR: 0.82) for AQP1, AQP2, AQP4, and AQP9, respectively. Patient baseline characteristics are presented in Table 1. The difference in patient age between the study groups turned out to be statistically significant. In cSDH, age was significantly higher compared to the group of patients with aSDH (*p* < 0.0001). Additionally. GCS values were significantly higher in the group of patients with cSDH compared to patients from the aSDH group (*p* = 0.005). Furthermore, the hematoma density was significantly higher in the aSDH group than in the cSDH group (*p* < 0.0001).

### 2.2. Plasma Concentrations of AQPs in Acute SDH 

**Figure 2:**
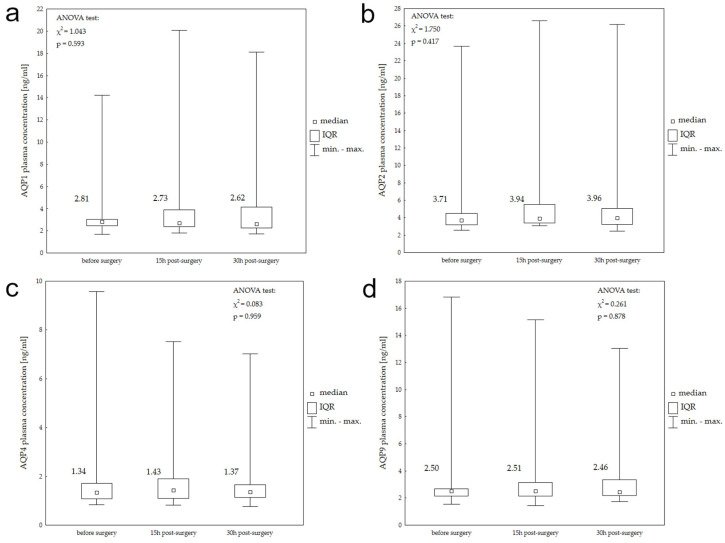
Plasma AQP1 (**a**), AQP2 (**b**), AQP4 (**c**), and AQP9 (**d**) concentrations before surgery, 15 h post-surgery, and 30 h post-surgery in patients with aSDH.

Figure 2 presents the median AQP1, AQP2, AQP4, and AQP9 plasma concentrations in the aSDH study group before, 15 h, and 30 h after surgery, and the results of the Friedman ANOVA test for each aquaporin. In the aSDH study group, none of the aquaporins showed significant differences between their serum levels at measured time points.

The results of Spearman’s correlation between plasma aquaporin concentrations and radiological features (hematoma volume, midline shift) of aSDH are presented in Table 2. In this study group, no statistically significant correlation was found between aquaporin plasma levels and hematoma radiological characteristics for AQP1, AQP2, AQP4, and AQP9.

### 2.3. Plasma Concentrations of AQPs in Chronic SDH

**Figure 3:**
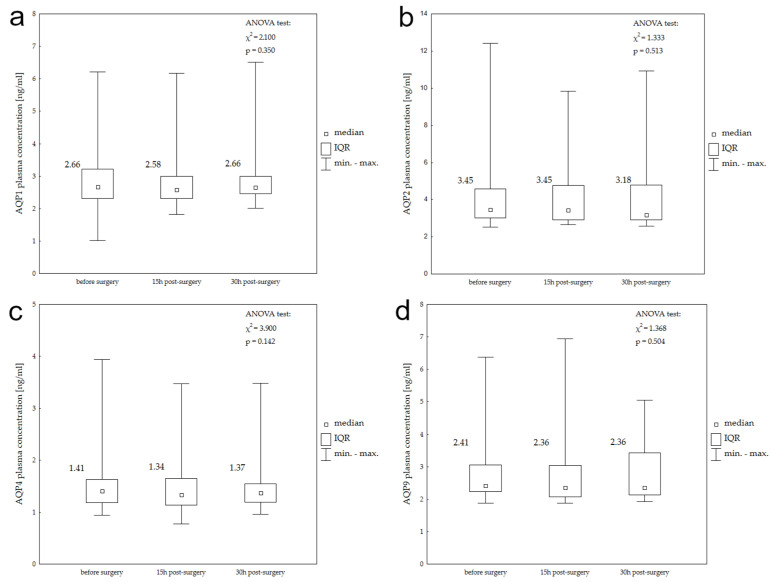
Plasma AQP1 (**a**), AQP2 (**b**), AQP4 (**c**), and AQP9 (**d**) concentrations before surgery, 15 h post-surgery, and 30 h post-surgery in patients with chronic SDH.

Median AQP1, AQP2, AQP4, and AQP9 plasma concentrations in the chronic subdural hemamotma (cSDH) study group before, 15 h, and 30 h after surgery with the results of the Friedman’s ANOVA test are presented in box plots (Figure 3). According to Friman’s ANOVA tests, none of the aquaporins demonstrated significant differences between their serum levels at the studied time points.

Table 3 shows the results of Spearman’s correlation between plasma aquaporin concentrations and radiological features of cSDH. Among the four studied aquaporins, statistically significant correlations were found only for AQP2 in the cSDH group. AQP2 plasma concentration correlated negatively with midline shift measured before surgery (Spearman’s R −0.54; *p* = 0.017) and positively with hematoma volume change between baseline and 30 h post-surgery (Spearman’s R 0.627; *p* = 0.007).

## 3. Discussion

The conventional approach to monitoring traumatic brain injury patients involves neurological assessments, clinical evaluations such as the Glasgow Coma Scale, and radiological scans, typically through CAT scans [6,7]. 

A test utilizing blood-based biomarkers has the potential to improve patient stratification, leading to decreased reliance on imaging, prediction of outcomes, early identification and management of secondary complications, and monitoring of treatment efficacy and overall healthcare quality [4].

Among the known particles useful in TBI, serum GFAP levels predict brain lesions on head CT, aiding triage. S100B, when used cautiously with clinical factors, refines the need for CT. NF-L levels on the day of injury predict incomplete mTBI recovery [18,19,20]. One of the molecules whose role is being analyzed in the context of monitoring the aftermath of central nervous system injuries is aquaporins. Among them, those most frequently studied and associated with various brain diseases are known as brain aquaporins, specifically AQP4, AQP1, and AQP9. AQP1 and AQP4, which are highly permeable to water, contribute to cytotoxic brain edema, while AQP9, with lower water permeability, plays a role in energy metabolism [21].

Numerous studies investigate the function of AQPs in TBI, with most focusing on the role of AQP4, which is predominantly found in astrocytes, particularly concentrated in their endfeet, facilitating bidirectional water movement in response to changes in osmotic conditions [12]. Fuqiang Guo’s team established a distinct correlation between AQP4 mRNA expression and the onset of secondary brain edema and damage following injury. The expression of AQP4 mRNA within brain tissue gradually rises post-trauma, peaking during the 12–72 h window, aligning with the peak of pronounced brain edema and cellular damage. These observations lead to the hypothesis that AQP4 could potentially function as a biomarker for evaluating the severity of cerebral edema and the extent of traumatic brain injury [22]. Another study, conducted by Yue Shi et al., investigated the potential of AQP4 as a biomarker for evaluating hematoma expansion in patients with traumatic intracerebral hemorrhage (ICH). The study revealed that while the expression levels of AQP4, MMP2, and MMP9 gradually increased over the first 24 h post-hemorrhage, dystroglycan expression decreased. Notably, MMP9 expression at 6 h post-bleeding onset correlated with hemorrhage severity, whereas AQP4 levels did not correlate with early hematoma enlargement. These findings suggest that while AQP4 may have utility in assessing cerebral hemorrhage, its efficacy as an early marker for hematoma expansion remains uncertain. Further research is necessary to fully elucidate the role of AQP4 as a diagnostic and prognostic marker in traumatic brain injury [23]. 

Similar observations were made regarding aquaporin 9 [13]. Research conducted on laboratory animals suggests that elevated levels of AQP9 could aid in removing excess water and lactate during the initial phase of TBI. The presence of abundant AQP9-positive astrocytes may facilitate the movement of lactate into neurons, potentially leading to cellular brain edema during the later stages of TBI. Additionally, the identification of AQP9-positive neurons implies a role for AQP9 in maintaining energy balance following TBI [24].

The importance of aquaporin 1 in the course of trauma was also analyzed [9]. Bo Qiu observed significantly elevated levels of AQP-1 expression in the hippocampi of mice post-TBI compared to the sham group, with peak expression occurring one day after injury. These findings suggest that increased AQP-1 expression may contribute to edema formation and delayed hippocampal cell death following TBI [25].

Moreover, increased expression of AQP1 has also been documented within the external capsule of chronic subdural hematoma, and it is known that it contributes to fluid accumulation and volume expansion of its growth [26,27]. 

While much attention has been given to other aquaporin isoforms, the specific roles and contributions of AQP2 in TBI remain largely unexplored. Similarly, during the planning of this research, our initial understanding of brain aquaporins guided the formulation of hypotheses and the study protocol. Consequently, we selected AQP2 as the control group, assuming its level in the blood would remain constant. However, our assumption that only brain aquaporins would alter their expression after TBI was incorrect. Contrary to expectations, levels of aquaporins AQP1, AQP4, and AQP9 did not exhibit significant changes in response to traumatic brain injury. However, in the group of patients with chronic subdural hematoma, a strong, positive correlation was found between the change in the volume of the hematoma due to surgery and changes in the expression of aquaporin 2. According to the results obtained, with the decrease in the volume of the hematoma after surgery, the concentration of aquaporin 2 in peripheral blood also decreased statistically significantly. This observation suggests a potential role for aquaporin 2 in the regulation of fluid dynamics during the resolution of subdural hematoma, warranting further investigation into its pathophysiological implications.

AQP2 plays a crucial role in the body’s water balance by facilitating the movement of water molecules from the urine back into the bloodstream [10,11,28]. The reabsorption of AQP2 into the bloodstream is primarily regulated by the hormone vasopressin, also known as antidiuretic hormone (ADH). Vasopressin, synthesized in the hypothalamus and secreted by the posterior pituitary gland, responds to alterations in blood osmolality or volume. During conditions necessitating water conservation, such as dehydration or reduced blood volume, vasopressin secretion escalates. Upon binding to specific receptors on collecting duct cell surfaces in the kidney, vasopressin initiates intracellular signaling cascades, prompting the translocation of AQP2-containing vesicles to the apical membrane of these cells [29]. Subsequent insertion of AQP2 into the apical membrane facilitates increased water reabsorption from urine into cells, and subsequently, into the bloodstream. This process aids in urine concentration and water conservation. Consequently, the reabsorption of AQP2 into the bloodstream is intricately regulated by vasopressin-mediated signaling pathways within the kidney, which are crucial for maintaining water balance and preventing dehydration [30]. The latest scientific research also confirms the presence of its isoforms in the peripheral nervous system [31]. 

Up until April 2022, the existing literature had not documented the occurrence or function of AQP2 in the human central nervous system. However, Deng et al. recently published a study examining the association between AQP2 levels in the brains of laboratory animals with induced non-traumatic intracerebral hemorrhage and plasma levels in patients with spontaneous non-traumatic intracranial hemorrhage (ICH), alongside their clinical prognosis. Moreover, this study identified AQP2 for the first time in glioma cells, astrocytes, and microglial cell lines from laboratory rodents. Additional research conducted on rats with collagenase-induced ICH demonstrated heightened AQP2 expression in astrocytes surrounding the hematoma area. These findings suggest a potential involvement of AQP2 in central nervous system processes related to extravasated blood and brain edema [32].

In the aforementioned study, conducted by Shuwen Deng and colleagues, data were gathered from 33 patients diagnosed with intracerebral hemorrhage (ICH), and serum levels of AQP2 were analyzed both in this patient group and in healthy individuals. A correlation analysis was performed between serum AQP2 levels and various clinical factors. The study revealed that patients with ICH exhibited lower serum AQP2 expression compared to healthy controls. Furthermore, lower serum AQP2 levels were inversely associated with 90-day modified Rankin Scale scores following ICH, although no correlation was observed with National Institute of Health Stroke Scale (NIHSS) scores upon admission. In a rat model of ICH, dual fluorescence staining of glial fibrillary acidic protein (GFAP) and AQP2 was conducted to explore the relationship between astrocytes and AQP2. This analysis revealed overexpression and localization of AQP2 within GFAP-labeled astrocytes in rats.

The results clearly demonstrated that conditioned media from astrocytes overexpressing AQP2 led to increased levels of CD86 and CD68 in microglia. Microglia and macrophages can be categorized into two phenotypes: the M1 phenotype, characterized by CD86 and CD68, among others, is considered pro-inflammatory due to its production of inflammatory cytokines, whereas the M2 phenotype, represented by CD206 and CD36, is anti-inflammatory [33]. According to the authors’ hypothesis, overexpression of AQP2 triggers astrocyte activation and pro-inflammatory effects through the TLR4/NFkB-65 signaling pathway. Furthermore, AQP2 indirectly promotes microglial polarization towards the M1 phenotype in the immortal rat cell line (HAPI), potentially amplifying the pro-inflammatory response following ICH. These findings suggest that AQP2 expression correlates with the intensity of the inflammatory response post-ICH and, consequently, with secondary CNS damage. Additionally, conditioned media from AQP2-silenced astrocytes resulted in reduced CD86 and CD68 levels in microglia, along with a decrease in the M1/M2 phenotype ratio. From these observations, it was inferred that AQP2 could potentially function as a biological marker of inflammation in serum during the early stages of ICH. Additionally, considering the pro-inflammatory role of AQP2 in glial cells, the authors hypothesized in the cited work that decreased AQP2 expression in peripheral blood serum might indirectly signify reduced AQP2 excretion, while increased AQP2 expression in peripheral blood cells or the CNS could be correlated with the worsening of neurological conditions in patients post-ICH.

However, the findings of the presented study diverge from expectations. Patients showing a reduction in the volume of chronic subdural hematoma, suggestive of a more favorable prognosis, exhibited lower concentrations of AQP2 in their peripheral blood samples. Conversely, it is widely accepted that a more severe disease state correlates with increased expression of AQP2 in the central nervous system. Despite this disparity, both studies underscore the significance of AQP2 in the inflammatory processes occurring within the central nervous system following intracranial bleeding.

Therefore, despite the lack of other reports in the available literature, AQP2 plays a more important role in the pathogenesis of chronic subdural hematoma and intracerebral bleeding than previously thought. Currently, considerations about the role it plays in these pathological processes take a hypothetical form. It is known that the external capsule surrounding the CSDH has been identified as the source of fluid exudate and bleeding. Angiogenic factors lead to the formation of fragile blood vessels in the capsule, and fibrinolytic processes prevent the formation of clots, which results in recurrent bleeding [34]. Many inflammatory cells and markers have been identified in the membranes and subdural fluid and likely contribute to the spread of the inflammatory response that stimulates continued capsular growth and fluid accumulation. The injury-induced inflammation process appears to be mediated by a number of inflammatory cells, including neutrophils, lymphocytes, macrophages, and eosinophils. However, the release of aquaporin-2 (AQP2) into the bloodstream triggered by inflammation involves complex molecular mechanisms that intersect with the regulation of AQP2 in the kidney. Inflammation initiates a cascade of events characterized by the release of various signaling molecules such as cytokines and chemokines. These molecules directly or indirectly influence AQP2 regulation in the kidney. Concurrently, inflammation can disrupt the synthesis and release of vasopressin, a key hormone that regulates AQP2 expression and trafficking in the kidney [35]. This disruption may affect the sensitivity of vasopressin receptors or interfere with the signaling pathways involved in vasopressin release [36]. Consequently, altered vasopressin levels impact the activation of adenylyl cyclase and the subsequent production of cyclic AMP (cAMP) within kidney cells [37]. Elevated intracellular cAMP levels activate protein kinase A (PKA), which in turn phosphorylates AQP2, facilitating its translocation to the apical membrane of collecting duct cells [38]. Additionally, inflammation may modulate the trafficking of AQP2-containing vesicles within collecting duct cells [39]. This modulation involves potential changes in the expression or activity of proteins associated with vesicle trafficking, including Rab GTPases, SNARE proteins, and motor proteins [40,41,42]. Furthermore, inflammatory signals influence the ubiquitination process of AQP2, which targets the protein for degradation or recycling. Changes in the activity of ubiquitin ligases or deubiquitinases under inflammatory conditions affect AQP2 abundance at the apical membrane of collecting duct cells [43]. Moreover, inflammatory conditions can alter the glycosylation status of AQP2, impacting its stability and trafficking [43]. Changes in osmolality associated with inflammation also play a role in AQP2 expression and localization in the kidney [44]. It may be hypothesized that similar mechanisms triggering the translation of AQP2 and the release of AQP2 protein into the bloodstream occur during neuroinflammation. However, this is speculative and requires further research.

The primary components of secondary damage following brain injury include, in addition to inflammation, oxidative stress, excitotoxicity, and cytotoxicity [45]. Tamma et al.’s study revealed that AQP2 expression is responsive to cellular reactive oxygen species (ROS) and intracellular calcium levels in kidney cells. Oxidative stress induced by reactive oxygen species from NADPH oxidase increases AQP2 transcription by inhibiting cAMP hydrolysis in the principal cells of the renal collecting duct [46]. Thus, it could be postulated that intracranial hemorrhage-induced oxidative stress and excessive neuroinflammation may lead to AQP2 overexpression in astrocytes and enhance AQP2 transcription.

In our investigation of paracerebral hematomas, we found a correlation between the reduction in hematoma volume within 30 h post-surgery for chronic subdural hematomas and the decrease in AQP2 concentration in peripheral blood serum. Conversely, Deng et al.’s study associated lower AQP2 concentration with poorer 90-day survival outcomes on the modified Rankin Scale (mRS). The authors did not consider radiological markers, which were the primary comparative element in our study. However, when comparing both studies, it’s important to consider the different dynamics of intracranial bleeding development: intracerebral bleeding is acute, while chronic subdural hematoma progresses over weeks, with symptoms appearing relatively late. Consequently, although both conditions likely involve increased AQP2 expression in nervous tissue, their expression in peripheral blood may exhibit opposite dynamics.

The authors of the study propose that the demonstrated relationship suggests a potential role for AQP2 as a biomarker of TBI. However, further research is needed to consider additional variables such as blood morphotic parameters and the level of inflammatory cells measured in peripheral blood, as well as examination of cerebrospinal fluid to analyze AQP concentrations, and AQP analysis of hematoma material obtained intraoperatively. Although the current study focused on the acute postoperative period, future steps should involve assessing the dynamics of aquaporins over a longer observation period of 10–14 days. These findings significantly contribute to the existing literature and open new avenues for research on this aquaporin in relation to central nervous system pathologies.

As we delve deeper into the role of AQP2, it becomes increasingly apparent that understanding its function and regulation may offer valuable insights into the mechanisms underlying cerebral edema and secondary brain injury following trauma. Thus, a comprehensive examination of AQP2 in the context of TBI holds promise for advancing our understanding of this complex condition and may lead to the development of novel diagnostic and therapeutic approaches.

## 4. Materials and Methods

### 4.1. Study Population and Eligibility Criteria

This prospective study included patients hospitalized in Independent Public Clinical Hospital No. 4 in Lublin, Poland, and the Department of Neurosurgery at the Mazowiecki Specialist Hospital in Radom, Poland, receiving surgical treatment for acute or chronic SDH between December 2018 and November 2020.

Study inclusion criteria were as follows: (a) patients after craniocerebral injury aged between 32 and 94 years old; (b) aSDH or cSDH diagnosed on CT scan; (c) surgical management by craniotomy, single or double burr hole surgery. In the case of acute SDH, a craniotomy or craniectomy was conducted. Whereas chronic SDH has been diagnosed, single or double burr hole surgery has been performed. The exclusion criteria were as follows: (a) diffuse traumatic brain injury; (b) penetrating head injury; (c) coexisting neurological disease, and (d) brain death diagnosed. Qualified study participants have been divided into two groups—patients with aSDH (aSDH group) and patients with cSDH (cSDH group).

In light of extensive research demonstrating the most pronounced alterations in AQP expression within the initial 48 h following injury, the decision was made to assess the concentration of aquaporins at three junctures: preoperatively, and at 15 and 30 h subsequent to surgical intervention.

Collected clinical data included (a) patient age at presentation, (b) presence of speech disorders, (c) presence of paresis, (d) midline shift value on CT scan, (e) density of hematoma on CT scan, (f) hematoma volume before, 15 and 30 h after surgery, and (g) consciousness level in Glasgow Coma Scale (GCS) in equal time intervals.

The study protocol was approved by the Bioethics Committee of the Medical University of Lublin (approval number: KE-0254/25/2018). All data were recorded anonymously.

### 4.2. Blood Sample Collection, Processing, and Storage

Two milliliters of blood plasma samples were collected from the study participants at three time intervals: the first sample during preparation for surgery, the second sample at 15 h after surgery, and the third sample at 30 h after surgery.

Ten milliliters of peripheral blood from the basilic vein have been collected from each study participant in tubes treated with EDTA. Then, samples were cooled to a temperature of 4 °C and immediately transported to the Laboratory of Biostructure of the Human Anatomy Department (Medical University of Lublin, Poland). Plasma was separated by centrifugation of blood samples at 2800× *g* for 5 min and finally stored in Eppendorf tubes (in the amount of 200 μL per tube) at −20 °C for 24 h and then at −80 °C until further use.

### 4.3. AQP Concentration Measurements

The plasma concentrations of AQP1, AQP2, AQP4, and AQP9 were determined by sandwich ELISA using a Quantitative Sandwich ELISA kit (My BioSource, Inc., San Diego, CA, USA) for each aquaporin. Analysis was performed using a Victor X4 multilabel plate reader (PerkinElmer, Inc., Shelton, CT, USA) in strict accordance with the manufacturer’s instructions. WorkOut software (ver. 2.5, Dazadaq Solutions Ltd., Lublin, Poland) was used to analyze the obtained data.

### 4.4. Radiological Examination

CT scans were performed on all patients qualified for the study before and after surgical treatment. CT images obtained in DICOM files were processed using the open-source software Horos (ver. 3.3.0, Horos Project). Then, density, maximal diameter of hematoma, and midline shift value have been calculated. 3D Slicer software was used for the 3D conversion of DICOM files to calculate the volume of hematoma before and after surgery.

### 4.5. Statistical Analysis

The collected data were analyzed descriptively and comparatively, and correlations between variables were calculated. Distribution analysis of the studied variables has been conducted using the Shapiro–Wilk test. Descriptive statistics of variables with a non-normal distribution were presented using the median and interquartile range (IQR). The Mann–Whitney U test was used for non-normal distribution variables to see whether there was a significant difference between groups.

The significance of the differences between the samples was assessed based on a non-parametric Wilcoxon test for related samples. A Friedman ANOVA test was conducted to explore the statistical significance of differences between AQP plasma levels measured before, 15 h, and 30 h post-surgery. Relationships between variables were examined using Spearman’s nonparametric rank correlation coefficient. The results are presented in the form of tables and figures.

All statistical analyses were carried out at a 5% level of significance. A *p*-value under 0.05 was considered significant. Microsoft Office Excel software (Microsoft Corp., Redmond, WA, USA) has been used for data collection. All statistical analyses have been conducted using the Statistica software (ver. 13.3, TIBCO Software Inc., Palo Alto, CA, USA).

## 5. Conclusions

In conclusion, the above study presents conclusions that are relevant to the nature of the subject under examination, showing a high correlation between the concentration of aquaporin 2 and the volume of the chronic subdural hematoma as well as midline shift, while indicating the lack of correlation between the concentrations of aquaporins 1, 2, 4, and 9 before and after the evacuation of the acute subdural hematoma and aquaporins 1, 4, and 9 after surgery for chronic subdural hematoma, as well as the patient’s clinical condition in the above clinical situations, assessed in GCS. The presented correlation between changes in the volume of extracerebral hematomas measured by radiological techniques and the concentration of AQP2 measured in serum seems to be the most important finding of the above work, making a key contribution to the development of research on determining the value of aquaporins as clinical indicators.

## Figures and Tables

**Table 1:** Baseline characteristics of the 41 study participants.

Variable	Total (*n* = 41)	aSDH Group (*n* = 21)	cSDH Group (*n* = 20)	*p* *
Demographic characteristics				
Age, years				
mean (SD)	65.9 (14.3)	58.6 (14.8)	73.2 (9.4)	**<0.0001**
median (IQR)	64 (19)	57 (12)	74 (9.5)	
Sex				
male	31	17	14	0.717
female	10	4	6	
Clinical characteristics				
GCS, median (IQR)	14 (3)	12 (9)	14.5 (2)	**0.005**
presence of paresis, *n*	24	15	9	0.061
presence of anisocoria, *n*	7	7	0	-
speech impairment, *n*	22	14	8	0.121
Radiological features, median (IQR)				
hematoma volume, cc	124.4 (90.2)	123.4 (84.4)	125.3 (90.2)	0.223
midline shift, mm	8.8 (8)	8.5 (5.7)	9.5 (6.1)	0.940
hematoma density, HU	40 (29)	61 (9)	32.5 (12)	**<0.0001**
Aquaporin plasma concentration, median (IQR)				
AQP1, ng/mL	2.81 (0.79)	2.82 (0.79)	2.60 (0.91)	0.494
AQP2, ng/mL	3.60 (1.38)	3.79 (1.45)	3.45 (1.21)	0.452
AQP4, ng/mL	1.39 (0.56)	1.38 (0.57)	1.39 (0.45)	0.847
AQP9, ng/mL	2.47 (0.82)	2.50 (0.76)	2.40 (0.68)	0.452

Abbreviations: SD—standard deviation; IQR—interquartile range; SDH—subdural hematoma; GCS—Glasgow Coma Scale; cc—cubic centimeters; mm—millimeters; HU—Hounsfield units; and *p* *—Mann–Whitney U test (for quantitative data) or χ^2^ test (for qualitative data). Baseline characteristics of the 48 study participants.

**Table 2:** Correlations between AQP1, AQP2, AQP4, and AQP9 plasma concentrations and radiological characteristics of acute SDH before surgery, 30 h post-surgery, and change in the variables.

Variable	AQP1			AQP2			AQP4			AQP9		
	*n*	R	*p*	*n*	R	*p*	*n*	R	*p*	*n*	R	*p*
Before surgery												
Hematoma volume	21	−0.327	0.148	21	−0.161	0.486	21	−0.446	0.054	21	−0.326	0.149
Midline shift	21	−0.010	0.964	21	−0.196	0.395	21	0.213	0.355	21	0.052	0.823
15 h post-surgery												
Hematoma volume	21	−0.034	0.884	21	−0.104	0.654	21	−0.028	0.904	21	−0.157	0.508
Midline shift	21	0.174	0.451	21	−0.025	0.913	21	0.093	0.690	21	−0.008	0.972
Post-surgery												
Hematoma volume	20	−0.068	0.777	21	0.209	0.364	21	−0.010	0.964	21	−0.012	0.958
Midline shift	20	0.184	0.437	21	0.112	0.628	21	0.068	0.770	21	−0.003	0.991
Change												
Hematoma volume	20	0.120	0.613	21	0.330	0.144	21	−0.130	0.575	21	−0.068	0.771
Midline shift	20	−0.036	0.880	21	0.169	0.464	21	0.073	0.752	21	0.130	0.574

*n*—sample size; R—Spearman’s rank correlation coefficient; *p*—significance level for Spearman’s coefficient.

**Table 3:** Correlations between AQP1, AQP2, AQP4, and AQP9 plasma concentrations and radiological characteristics of chronic SDH before surgery, 30 h post-surgery, and change in the variables.

Variable	AQP1			AQP2			AQP4			AQP9		
	*n*	R	*p*	*n*	R	*p*	*n*	R	*p*	*n*	R	*p*
Before surgery												
Hematoma volume	20	0.023	0.925	19	0.193	0.429	20	0.215	0.363	19	0.158	0.519
Midline shift	20	−0.283	0.226	19	−0.540	0.017	20	0.082	0.731	19	−0.141	0.566
15h post-surgery												
Hematoma volume	20	0.050	0.835	19	−0.062	0.800	20	−0.213	0.368	20	−0.143	0.548
Midline shift	20	0.056	0.814	19	−0.112	0.647	20	0.070	0.770	20	0.049	0.837
Post-surgery												
Hematoma volume	20	−0.183	0.441	19	<0.001	1.000	20	−0.237	0.315	19	−0.136	0.567
Midline shift	20	0.106	0.656	19	−0.098	0.689	20	0.003	0.990	20	0.330	0.156
Change												
Hematoma volume	19	−0.168	0.491	17	0.627	0.007	19	0.302	0.209	18	0.391	0.108
Midline shift	19	−0.374	0.115	17	0.174	0.505	19	−0.118	0.631	18	−0.143	0.572

*n*—sample size; R—Spearman’s rank correlation coefficient; *p*—significance level for Spearman’s coefficient.

## Data Availability

Data are contained within the article.
